# Medical and Physical Intelligence

**Published:** 1820-04

**Authors:** 


					JWeMtal anil Intelligence.
JgOYAL SOCIETYNovember 11 th.?The conclusion of a paper
was read, detailing the results of some further researches of Sir
Everard Home on the properties of the blood. He has, he considers,
discovered that there exist in that fluid globules which are smaller and
of a different nature from those it is commonly supposed to contain.
They were first observed by Mr. Bauer, whilst examining the layers
composing an aneurismal tumor. They were seen in the coat in con-
tact with the circulating blood, in the proportion of one to four, com-
pared with the larger globules; but in the other layers they were more
numerous, and in that which have been first formed they existed in the
proportion of four to one. Mr. Bauer estimates their size of
an inch. Crystals of muriate and phosphate of soda, and phosphate
of lime, were found in making a section of another aneurismal tumor.
Sir Everard Home considers that those globules existed originally in
the serum ; the globules being to be seen only after the blood has coa-
gulated. In coagulated lymph, formed during intense inflammation,
these globules were found mixed with a few colourless blood-globules.
2;*
Mtdical and Physical Intelligence. 345
They were also found in great numbers in the upper firm coat of the buff
of the blood, while the lower and softer parts consisted chiefly of red
blood-globules. He proposes to call the new globules by the name of
globules of lymph, to distinguish them from the red blood-globules. Sir
Ererard Home has also found, that the quantity of carbonic acid gas
evolved from buffy blood under an exhausted receiver, is much less
than that from healthy blood, and that by far the greatest quantity of
this gas was yielded by blood drawn from a healthy person an hour
after a full meal. Both lymph globules and blood globules wero
found in the mucus of the pylorus and duodenum. In chyle, the size
of the globules is various.
Royal Academy of Sciences of Paris. Sitting of Jan. 3, 1820.?-
A memoir by M. Geoffroy Saint-Hilaire was read, containing some
views respecting the organization of Insects, of which we shall endea-
vour to give our readers some idea in an abstract. M. St.-Hilaire,
indeed, confines himself almost absolutely to general statements in the
present memoir; the particular applications of his notions being de-
ferred until future occasions.
M. St.-Hilaire considers that insects are vertebral animals, differing
from those generally so termed chiefly in this point of view, in the
skeleton being external instead of internal to the muscles, &c. The
following are the principal circumstances on which those notions are
founded.
Insects, in general, may be readily parted longitudinally into six
segments.
A first y which has hitherto been called the head.
A second, named the corselet. It may be best designated by stat-
ing it to be that portion to which the first pair of legs is always
attached.
A third, the principal character of which is to furnish a basis for
the insertion of the first pair of wings : generally called the scutellum.
A fourth, or the thorax properly speaking, which commonly sup*
ports the four hinder legs and the second pair of wings.
A fifth, composes the abdomen.
The sixth, and last, is composed of the anal ring, and often has se-
veral appendages, *
The first segment of the body of insects corresponds not to the
whole head of the vertebral animal, but to the bones of the face, of
those of the cerebrum,* and the hyoides.
The second answers to the bone enveloping the cerebellum,* those
of the palate and larynx.
The third is composed of the interparietals, parietals, and the
operculars.
Thus the three anterior segments arise from a separation of the
cranium of vertebral animals.*
* The reader will find some elucidation of those points in the Number of this
Journal for July 1819, and in the second article in that for January 1820.
NO. 254. 2 y
346 Medical and Physical Intelligence.
iThe three segments which follow correspond to the trunk of the
latter, and are, like that, divisible into thorax, abdomen, and coccyx.
The posterior wings are analogous to the swimming-bladder of
fishes, or, what is the same thing, the air.celis of birds. At the in-
stant of the transformation of the nymph into a perfect insect, the
wing is a sort of vesicle; on becoming dry, the membranes fall into
contact, and then, apparently, form only one lamina.
Observations respecting the Pathology of Hooping-Cough.?We
usually comprise in the Report of Diseases, such of the chief results
of our practical observations of the current period as are of general
application ; but we are now induced to give, in a distinct manner, an
account of some which have just occurred to us on opening the body
of a subject of hooping-cough, from their seeming to possess a remark-
able degree of interest.
A boy, three years old, had suffered hooping-eough about a month,
not very severe in degree, and unattended with much difficulty of re-
spiration, excepting for a short time after each fit of coughing. He
was a strong, well-made child, and had previously enjoyed good health.
At the time above indicated, he became affected with great difficulty of
respiration, and very severe cough, attended with extraordinarily loud
hooping, and long-continued inspiratory efforts. The pulse varied
during the two or three ensuing days from 120 to 150; the tongue was
clean. There was a moderate degree of expectoration of mucous
matter. Leeches, tartar emetic, calomel, and a blister on the chest,
had been employed by the attending medical practitioner, Mr. Rankin,
of Marchmont-street. On the fifth day after the occurrence of the
latter symptoms, in the evening, we first saw the patient. But little
hopes of his survival were entertained. The pulse was 170, mode-
rately strong; the countenance somewhat tumid and livid; the breath-
ing extremely laborious, and attended with a rattling sound similar to
that heard in croup. The nostrils were widely dilated during inspiration.
The fits of coughing were said to be very severe, and attended with
intense and long-continued hooping ; and the mother said, it appeared
as if the air was prevented being expelled from the lungs by some ob-
stacle, the dilatation of them was of such long duration. He died in the
ensuing morning. The following were the observations made on dis-
section, that seem to be peculiarly interesting.
The posterior surface of the epiglottis was of a palish bright-red
colour, especially towards its base; the right superior ligament of the
glottis was about four times the usual thickness, apparently from in-
flammation, and its tunic was of a bright-red colour ; the left superior
ligament was much smaller, apparently only about two or three times
the usual size; they both adhered to the lower ligaments, so as to
efface the openings into the ventricles of the larynx by false membranes.
The ventricles themselves were full of serous fluid. The lower liga-
ments were also much thickened, and their mucous tunic of a florid
hue. Below this, the whole of the larynx was apparently healthy.
At about the third or fourth circle of the trachea, the mucous mem-
brane of this organ began to assume a florid hue, and there was extra-
1
Medical and Physical Intelligence. 347
vasation of blood in it, bat no coagulated lymph present. These ap-
pearances increased in intensity as we proceeded downwards, and the
two lower lobules of the left, and the lower lobule of the right, lung,
were in a state of extreme sanguineous congestion. The enveloping
pleura was of nearly the natural hue, and there was no preternatural
effusion of fluid in the chest. The mucous membrane of the bronchia*
corresponding to these portions of the lungs was of a very deep-red
colour, and much thickened from the increased vascularity, but there
was no appearance of effused coagulable lymph. Every thing indicated
that the affection of the trachea and lungs was of recent date; whilst
that of the parts about the glottis was evidently more chronic, and
wholly distinct from the former.
It would be wrong to generalize the application of these ob-
servations ; but we hope they will induce our readers to examine very
particularly the state of the upper part of the laryux in the subjects of
hooping-cough ; and we should feel much gratified, if any of them will
favour us with the results of their observations.
Bills of Mortality, fyc,for London, of the Ytar 18ip.
DISEASES.
Abscess .........
Apoplexy .......
Asthma ?????????<
Bedridden
Cancer .........
Childbed   229
Consumption ......
Convulsions
Croup
Diarrhoea
Dropsy
Dropsy in the Brain .
Dropsy in th? Chest
Dysentery
Epilepsy ?
Eruptive Diseases ? ?
Erysipelas, or St. An-
thony's Fire
Fever ????????????
Fever (Typhus) ..
Fistula
Flux
Gout
Haemorrhage ......
Hooping-Cough
Hydrophobia ......
Inflammation ?????<
Inflammation of the
Liver  71
Insanity ????? 240
Jaundice    41
Measles   695
Miscarriage ...... 3
Mortification ?????? 399
Old Age and Debility 1850
Palsy ............
Venereal ........
Rheumatism ......
Rupture
Scrofula
Small-pox
Sore Throat and Quin-
sey  19
Spasm    42
Still-born ???  673
Stone  24
Stoppage in the Sto-
mach    18
Suddenly    310
Teething   502
Thrush   118
Worms   8
CASUALTIIS.
Broken Limbs ? ? ? ? 1
Burnt   27
Drowned    97
Excessive Drinking 4
Executed ?   ^10
Found Dead ..???? 10
Fractured   %
Frightened ? 4
Killed by Falls and se-
veral other Accidents 65
Killed by Fighting ?? 1
Killed by Lightning 1
Murdered   2
Poisoned ........ 2
Scalded 2
Strangled ........ 1
Suffocated ???????? 2
Suicidcs ........... 35
Total .... 266
Christened In all, *4,300
B"-d ? fSes SsW J In'aI1' 19'??
Whereof have died:
Under Two Years of Age . . 4779
Between Two and Five . . 1771
Five and Ten ? . ? 8*6
Ten and Twenty . ? .631
Twenty and Thirty . . . 1577
Thirty and Forty . . . 1990
Forty and Fifty . . . 2095
Fifty and Sixty . . . 19 IB
Sixty and Seventy . . 1600
Seventy and Eighty . . 1230
Eighty and N-inety . . . 666
Ninety and a Hundred . . 144
A Hundred
A Hundred and One
A Hundred and Two
A Hundred and Three , ? 1
Decreased in the Burials this year, 477.
2 Y 2
348 Medical and Physical Intelligence.
Prize Questions.?The following is proposed by the Society of Sci-
ences of Harlem. The essays should be sent to the Secretary before the
1st of January, 1821.
" What advantages has medicine derived from the reformation and
extension of chemistry since the time of Lavoisier, in making us better
acquainted with the chemical agency of the medicines usually employ-
ed for the cure of several diseases of the human body ; and what means
should be taken in order to acquire a solid knowledge, useful in
medicine, of the hitherto-unknown chemical agency of several medi-
cines."
The Medical Society of Paris proposes the following:
" To determine the nature, the causes, and the treatment, of the
convulsions which occur during pregnancy, in the course of parturi-
tion, and after delivery
The memoirs, written in L'atin or French, bearing an epigraph re-
peated in a sealed billet, which shall contain the name, quality, and re-
sidence, of the author, should be sent, free of expense for carriage, to
the Secretaire Gen b ale^ before the 31st of October, 1820. The prize
is 300 francs.
New Mode of treating Navi Materia.?Several cases of this affec-
tion have lately been successfully treated in Germany by inoculating
the part with vaccine virus.
A child had on the right cheek a naevus of a deep-red colour, about
a quarter of an inch in diameter. Some vaccine virus was introduced
under the cuticle of the affected part: on the ninth day, a very large
vesicle had formed, and the surrounding inflammatory areola covered
almost the whole of the cheek. At the end of a month, the naevus had
disappeared: a slight mark of a pale reddish colour only remained.
A naevus of the size of a shilling, of a deep-blue colour, situate near
the axilla, was inoculated with vaccine virus by Dr. Burkhardt: eight
vesicles formed, suppuration became established, and the naevus disap-
peared.
Six cases of naevus were treated in the same way by Dr. Klein. In
the greater proportion of them, a red areola appeared, which conti-
nued until the desiccation of the pustules; on the falling off of the
crusts,. a regular cicatrix was discovered, and the naevi had disappear-
ed. Dr. Muller has seen this mean successfully used in nine cases.
M. Lapostolle, professor of chemistry and physics at Amiens, has
endeavoured to prove, by some ingenious experiments, that the best-
constructed metallic conductors are far from being perfect con-
ductors of lightning ; that those which have been broken, or which
are not thrust very deep into the ground, are still more imperfect;
that the facts which have hitherto been alleged to be calculated to es-
tablish the property of transmitting electricity attributed to iron,
demonstrate the ce attraction" of the metal for it rather than its con-
ductibility; lastly, that straw is the conductor par excellence: " its
power in this respect is such, that we may with impunity expose our-
selves to the influence of a powerful apparatus, the hand being only
Medical and Physical Intelligence. 349
armed with a stalk of straw an incb in length, and thug sustain the
electric fluid without experiencing the slightest shock."
These experiments have been repeated with entire success by the
members of the Association de bienfaisance Medicate formed at Amiens,
for the treatment of atonic neuroses by means of electricity, and com-
posed of MM. Seigneur-Gens, Mitiffen, Griyis, and Trannoy. It is
from a note inserted in the Journal d'Agriculture et de Commerce of
the department of La Somme that we have taken the foregoing ex.
tract.
M. Lapostolle having conceived the hope of making this singular
property of straw serve as a mean of preventing the ravages of light-
ning, and even those of hail, proposes, in consequence, to raise on the
whole surface of our land poles of the linden-tree, or some other
light wood, about twenty feet in length, to serve as a support to a
band of straw surmounted by a metallic point. Only one apparatus
of this sort would be sufficient, he says, for a square containing sixty
acres, and its price will not exceed three francs.?Bibliotheque Medi~
caley December 18lp.
Statistical Observations on the Results of different Modes of treating
the present Epidemic Fever of Italy.?According to the statement of
Dr. Ottavia.ni,* it appears, on comprising the patients in the
hospitals with those in the city of Rome and its enTirons, (where
the fever is treated in all sorts of possible ways by different phy-
sicians,) that the proportion of the mortality to that of the patients
was as 28 to 100; and, in the practice of some particular physi-
cians, the proportion was as 46 to 100. But, in the hospital S.
Giovanni di Dio at Rome, where the contra-stimulant method was
employed, the proportion of deaths to the patients was only as 4 fo
100: that is, from the 1st of January to the 26th of July, there en-
tered 337 patients, of whom only 14 died.
As the strict veracity of all partizans of disputing sects is often
doubted, we shall add to the statement of Dr. Ottaviani the instruc-
tions circulated by the order of the Tribunale Sanitario delta Sacra
Consulta of the same city :
Article the 7th of the circular of the Supreme Government of Rome
of the 24th of May, 1817.?<c Wherever the antiphlogistic method of
treating the disease has been employed, the nosographic tables have
but rarely shown fatal results, or death, in a greater proportion than
7 or 8 in 100; whilst this proportion has risen to 40 under the ex-
clusively-stimulant method."
Another work before us presents some circumstances in the
difference of the results of the practice of the same physician, that
should make a deep impression on every medical practitioner. At
Vicenza, (where the epidemic has the same character as at Rome,) Dr.
Thiene makes it appear, + that, in his own private practice, he lost
37 in 100 ; in the Lazeretti, 40 in 100 ; in the prison, 13 in the same
number. Dr. T. though a Brunonian, fortunately, his adversaries
* In his Cenni sulla Febbre Petecchiale di Roma, nelV anno 1817.
t In his BUanci? Medico del Tifo che Regnio Epidemico sulla Provincia Vicentina
nel 1817. Vicenza.
350 Report of Diseases.
?will say, mingled, in spite of his intentions, contractimutants with hi#
excitants, as he used some saline medicines, belladonna, &c. as well as
trine, &c. But, what is the reason of the difference of the results of
his practice in the prison ? What does it show ?
REPORT OF DISEASES.
HAD any doubt remained respecting the greater prevalence of
disease in severe than in temperate winters in London, the pre-
sent would furnish decisive evidence against the notions somet ime
since entertained, of the healthiness of frosty and " bracing" wea-
ther. It is true, the form of fever called typhus has almost wholly
disappeared, and apoplexies are much more rare than they were during
last winter; but this alleviation is more than counterbalanced by the
extraordinary prevalence of very severe pulmonic affections, and rheu-
matism showing a great disposition to attack the heart in a serious
manner. Haemoptysis assumes the next place, in respect to frequency,
amongst the prevalent diseases of the season. On this we make but
one remark,?that many practitioners are disposed to rely too much
on simple depletory means, as blood-letting and low diet, without the
aid of revulsive measures. Large blisters on the arms or thighs will
occasionally be found to arrest the hemorrhage instantly, when blood-
letting has been employed in vain.
Hooping-cough, in an unusual severity of form, amongst children,
has also much swelled the list of severe and frequently-fatal maladies.
The latter has very commonly either commenced, or has become ac-
companied in its course, with inflammation of the lungs, which has in
a considerable majority of instances terminated in death. This great
fatality has however been chiefly observed in the lower classes of per-
sons, who are generally more ready to treat their children themselves
with qudck nostrums, than to resort to proper medical aid. In
many cases, the symptoms of inflammation of the lungs have not ap-
pealed until the second or third week of the disease; and it is remark-
able, that this affection has happened in several instances in children
who had been constantly confined to a warm nursery, ?nd who had
never, apparently, been exposed, from the time of the appearance of
the cough, to any considerable changes of temperature. Has the
great distension of the lungs, and the difficulty in expiration, any thing
t& do with the production of this ? Emetics of ipecacuanha, with the
occasional use of calomel, and in some cases with the addition of tartar
emetic, and a regular exhibition of a mixture composed of subcarbonate
of potash and extract of conium, have been apparently the most bene-
ficial remedies in this severe cpidemy. Amongst the small proportion
of those patients who have outlived pulmonic inflammation joined
with hooping-cough, several have continued to spit large quantities of
mucus, and a few muco-purulent, matter.
The catarrh of old age has also been very severe in general, as well
as unusually prevalent, and has sometimes terminated in anasarca. On
the occurrence of this, the expectoration has commonly ceased; and
in some of these cases the reproduction of the expectoration by squills
and ipecacuanha has btfen followed by a disappearance of the dropsy.
METEOROLOGICAL JOURNAL.
By Messrs. William Harris and Co. 50, Holborn, London.
From January 20 to February 19, inclusive.
OZ2
aS
Jan.
20
21*
22
23
24
25
26
27
28
29
30 O
31
Feb.
1
2
3
4
5
6
7
8
9
10
11
12
13
14
15
16
17
18
19
Rain
gauge
?02
?04
?03
?22
?24
TIIERM
33
33
30
33
39
44
*6
48
45
42
42
44
42
35
36
36
42
44
48
47
44
46
41
46
43
39
35
2
SO
29
34
34|31
35 26
3l'27
36 35
42*43
45,44
4745
50144
4940
47140
48'42
47 39
45 34
40:35
38 35
3939
4640
47 46
3426
35'32
35j30
BAROM.
29*61
29-30
30*09
30*10
29*84
29-87
29*56
29*60
49*60
30*20
SO-05
30"0o
30*00
29*87
30*00
30-09
30*02
29-90
30-17
30.15
30-07
30-00
30*10
29*88
30*02
29'25
29*70
30*21
30*04
29-90
29*47
29*67
29*40
30-04
30*15
30*07
30-10
29-90
29-93
30*06
30*10
29*93
30-09
30*20
30-16
30*03
30-13
29-28
29*05
30*10
45 36
41'34
36|28
35 28 30*24 30*17
30-25:30-26
30.32b0*32
30*1330*11
30*1230*10
50*1230-08
De Luc's
HYGROM.
Dry iL>amj
WIND.
NW
SW
NE
s w
ssw
s
w
ssw
NNE
K
wsw
wsw
s
ESE
E
WNW
S
w
sw
wsw
sw
W NW
SSW
w
S
NE
ENE
E
E
E
NE
ESE
NE
E
SW
WSW
s
sw
wsw
N
sw
sw
sjw
SSE
SE
N
SSW
SSW
wsw
wsw
MW
w
w
wsw
ESE
ESE
NE
NE
E
SE
NE
ENE
ATMOSPHERIC
VARIATION.
Fine
Cloud.
Fine
Rain
Cloud.
Cloud.
Rain
Fine
Fine
Cloud.
Rain
Cloud.
Fine
Cloud.
Cloud.
Cloud.
Cloud.
Sho'ry
Cloud.
Cloud.
Fine
Sleet
Cloud.
Cloud.
Cloud.
Cloud.
Cloud.
Foggy
Foggy
Fine
Foggy
Fine
Sleet
Cloud,
Rain
Fine
Cloud,
Rain
Fine
Rain
Fine
Cloud.
Fine
Fine
Fine
Fine
Cloud.
Sleet
Cloud.
Fine
Fine
Fine
Fine
Rain
Fin?.
Fine
Fine
Fine
Fine
Fine
Rain-gauge frozen.
t 362 ]
meteorological journal.
By Messrs. William Harris and Co* 50, Holborn, Londort*
\ From February 20 to March 19, inclusive.
?C2 d
"Is
QS 2
Rain
guage
Feb.
20
21
22
23
24
25
26
27
28
29
Mar
*1
2
3
4
5
6
7
8
9
10
11
12
13
14
15
16
17
18
19
THERM.
?07
?10
?06
?02
?04
?03
?12
?01
3233
32135
38
47
41
39
35
36
33
32
42
34
32
n
36
34
35
36
36
38
35
36
45
47
56
56
45
42
44
45
49
44
40
36
36
36
42
44
37
36
36
36
36
36
38
40
43
50
44
47
52
58
58
52
48
46
33
29
28
27
2?
27
34
31
34
32
34
38
39
BAROM.
29*87
29*^0
29-80
29-61
29-50
29*50
29 '96
30-13
30 02
29-90
29*96
29-80
29-80
29-55
29*44
29-70
30-12
30-10
29*95
29-69
29-53 29*57
29*00 29-48
29*76
30*03
30*20
30*19
30*19
30*26
30-20
29-86
29-66
29*50
29-70
49130-10
48 30*28
37
38
38
37
30*37
30*27
30*29
30*24
29*95
30-04
30*25
30-20
30*14
30*26
30*08
29-72
29-60
29*46
29-90
30-20
30-29
30*35
30*29
30-31
30*25
De Lut's
Hygrom.
Dry Damp
14
14
24
27
21
19
15
11
13
13
13
11
9
10
8
9
8
13
12
12
13
12
13
16
19
17
14
10
9
14
20
26
24
22
16
13
12
13
13
12
10
10
9
9
8
10
13
15
12
1*
13
14
18
18
15
12
10
9
WIND.
S
SSE
NE
S
wsw
NEN
ENE
ENE
ss*w
NNW
N
N
N
ENE
ENE
NNE
NE
WSW
s
SE
ESE
SW
SW
WNW
ESE
N
NE
NE
ATMOSPHERIC
VARIATION
E
1 NE
E
S
N
ENE
E
NE
E
WSW
WNW
var.
N
NNE
NE
NE
E
N
ESE
SW
S
ESE
ESE
NW
WSW
ESE
E
NE
NNE
E
Snow
Fine
Rain
Cloud.
Cloud.
Fine
Cloud.
Fine
Fine
Foggy
Clod .
Snow
Fine
Fine
Fine
Cloud.
fine
Fine
Foggy
Fine
Foggy
Fine
Fine
Fine
Fine
Cloud.
Sleet
Fine
Cloud.
Cloud.
Sleet
Rain
Rain
Sleet
Fine
Cloud.
Fine
Fine
Cloud.
Fine
Snow
Fine
Fine
Cloud.
Fine
Fine
Cloud.
Sleet
Cloud.
Sleet
Sleet
Rain
Fine
Cloud.
Snow
Sm.Sn.
Cloud.
Cloud.
Cloud.
Sleet
Foggy
Fine
The quantity of Rain fallen in February is BO'lOOth of an inch.

				

